# Fatal *α*-PVP and amphetamine poisoning during a sauna and autoerotic practices

**DOI:** 10.1007/s12024-020-00236-1

**Published:** 2020-03-26

**Authors:** Philippe Lunetta, Pirkko Kriikku, Julius Tikka, Ilkka Ojanperä

**Affiliations:** 1 Department of Biomedicine, Forensic Medicine, University of Turku, Turku, Finland; 2 Department of Forensic Medicine, University of Oulu, Oulu, Finland; 3 Finnish Institute for Health and Welfare, Forensic Toxicology Unit, Helsinki, Finland; 4 Department of Forensic Medicine, University of Helsinki, Helsinki, Finland; 5 Finnish Institute for Health and Welfare, Forensic Medicine Unit, Helsinki, Finland

**Keywords:** Autoerotic death, Sauna, Hyperthermia, *α*-PVP, Amphetamine, Contraction band necrosis

## Abstract

We describe the sudden death of a middle-aged man while having a sauna under the influence of *α*-pyrrolidinovalerophenone (*α*-PVP) (PM blood concentration: 0.8 mg/L), amphetamine (0.34 mg/L), and other drugs (buprenorphine, benzodiazepines), and engaging in solitary sexual activities. The drugs’ effects on the cardio-circulatory system and on body thermoregulation combined with the high temperatures are likely to have been central mechanisms leading to death. The high levels of adrenaline triggered by sexual arousal and the respiratory depression caused by buprenorphine, in association with benzodiazepines, may have also contributed to his death. This previously unreported type of accidental autoerotic death illustrates the risk of using amphetamine-like sympathomimetic drugs (e.g. cathinone derivates) in hot environments such as a sauna, and during sexual activities therein.

## Introduction

Deaths associated with autoerotic activities have been described in the medical literature for over a century [1]. Although several definitions and criteria for classification exist [1–4], autoerotic fatalities can merely be seen as “deaths that occur as the result of or in association with masturbation or other autoerotic activity” [1]. Byard and Bramwell [2] have recommended that the use of this term is restricted to accidental deaths that occur “during individual, usually solitary, sexual activity in which a device, apparatus, or prop that was employed to enhance the sexual stimulation of the deceased in some way caused unintended death”. The most frequent autoerotic accidental death (AAD) occurs by asphyxia, usually by hanging, and is the one frequently defined as typical [1, 5], but descriptions cover a wide range of atypical AAD [1, 3, 6–9].

We describe an atypical AAD of a middle-aged man during a sauna while under the influence of *α*-pyrrolidinovalerophenone (*α*-PVP), amphetamine, and other drugs.

## Case report

### Case history

A Caucasian male in his mid-40s was found dead, in late spring, in the sauna area of an apartment building, where he lived with his mother. The man had a history of multidrug abuse and was participating in a buprenorphine replacement therapy program (Suboxone®). He had been diagnosed also with attention deficit hyperactivity disorder **(**ADHD), for which his treatment was methylphenidate and diazepam. The victim’s mother had started an hour-long sauna-shift at 9:00 pm but returned to her apartment at 9:30 pm, at which time the victim began his sauna. After approximately midnight, the mother noticed that her son had not returned from the sauna. The sauna electrical heating system had shut off automatically at 10:00 pm and, simultaneously, the door to the sauna department was automatically closed by an electrical lock. At 1:48 am, the mother alerted the residence caretaker, who opened the sauna door at 2:30 am. The victim was found dead on the second highest bench of the sauna, in a supine position partially on his right side. He was unclothed except for a woman’s bra, which was placed around his hips. On the upper bench were a pornographic magazine and a wig. Beneath the benches were an anal dildo and two balloons that likely had been positioned under the victims’ bra.

No resuscitation attempts took place, as secondary signs of death were evident. At the time of police-scene investigation, around 3:00 am, the temperature in the sauna room was 43 °C. Police investigation revealed no suicide note nor any findings suggesting foul play.

### Autopsy findings

A medico-legal autopsy was performed 11 days later. At external examination, the victim’s body showed mild to moderate burn-like lesions caused by exposure to heat in the sauna. In addition to a few scattered bruises and abrasions, no other external signs of mechanical trauma were detectable. At internal examination, the brain and lungs showed congestion and moderate edema. Microscopic examination of sections of the left ventricular wall and ventricular septum showed myocardial contraction band necrosis and fragmentation of myocardial cells and dissection at the intercalated disks, chronic hepatitis and moderate hepatic fibrosis as well as, in the lungs, some foreign body granulomas consistent with intravenous drug abuse.

### Toxicological analysis

Post-mortem (PM) blood samples from femoral veins were positive for *α*-PVP (0.81 mg/L), amphetamine (0.34 mg/L), alprazolam (<0.005 mg/L; therapeutic range 0.01–0.02 mg/L), and oxazepam (0.18 mg/L; therapeutic range 0.1–1.4 mg/L) (GC-MS), as well as buprenorphine (8.7 μg/L; therapeutic range 0.5-10 μg/L), and norbuprenorphine (57 μg/L). In addition, traces of naloxone were detectable in PM blood, which is in accordance with the victim having been in buprenorphine replacement therapy. Amphetamine, *α*-PVP, and oxazepam were also detectable in the vitreous humor. No alcohols or other positive findings were detected either in blood, urine, or vitreous humor.

Based on circumstantial data, medical history, and autopsy findings, the cause of death was certified as “fatal poisoning by *α*-PVP and amphetamine” and the manner of death “unintentional”. “Effect of high temperature (hot air, sauna)” was deemed a contributing cause of death.

## Discussion

Cases of AAD are often classified as either typical (resulting from different types of mechanical asphyxia), or atypical (involving sexual self-stimulation by other means) [1]. The latter include electrocution, foreign-body insertion, intoxication, and even drowning [6, 7, 9–14]. A few cases of AAD related to or associated with cold exposure have been reported [1, 14]. Conversely, we are aware of only one AAD directly linked to environmental hyperthermia. That male victim was found dead outdoors (39 °C) wearing female clothes, including seven layered pairs of pantyhose, and had used a prescription medication (benztropine) causing, as a side-effect, hyperthermia [11]. Moreover, in some AAD cases, ones characterized by body-wrapping causing asphyxia, hyperthermia may have been a contributing factor [15]. Most often, PM toxicology in AAD is negative [8]. However, in addition to alcohol-positive cases and fatalities involving gas mixtures, some medicinal and illicit drugs have sporadically been detectable [5–9, 14].

In the present case, PM toxicology revealed *α*-pyrrolidinovalerophenone (*α*-PVP, *α*-pyrrolidinopentiophenone) in the blood in addition to amphetamine, buprenorphine, and benzodiazepines. The synthetic cathinone-derivate *α*-PVP, which is structurally related to pyrovalerone, acts as a central nervous system stimulant with effects similar to those of amphetamines and cocaine. Cathinone occurs naturally in a plant known as “khat” (*Catha edulis*), which has traditionally been chewed in Eastern Africa and in some Arab countries for its stimulant effects [16–18]. During recent decades, synthetic cathinones have been meant to replace more strictly regulated stimulants (e.g. cocaine, MDMA, other amphetamines) and to provide legal chemicals with equal or greater effects and abuse potential [19, 20]. Synthetic cathinones are commonly self-administered by tablet ingestion or powder insufflation, more rarely by intravenous or -muscular injection [19, 21]. Synthetic *α*-PVP has its peak effect within 10 to 40 min after intake, and its effects last for up to 1–3 h [18]. Users of synthetic cathinones report enhanced energy, euphoria, and empathy in social interactions, as well as increased libido [21–23]. Although perceived as relatively safe, these drugs have adverse sympathomimetic effects including arrhythmias, hypertension, hyperthermia, and seizures, as well as psychotic symptoms such as agitation and hallucinations [17, 19, 21, 22, 24].

Thus far, no data exist that can substantiate the toxic and lethal concentrations of *α*-PVP in humans [18, 19]. In the current case, the *α*-PVP PM blood concentration (0.81 mg/L) was fairly high compared to those concentrations detected in 47 individuals who underwent a medico-legal autopsy in Finland from January 2010 to September 2019 (*unpublished data*) and who tested positive for *α*-PVP in their blood (age range: 19–62, mean: 26.6 years, male-to-female ratio: 8.4). In this Finnish series (28 accidents, 9 suicides, 7 natural deaths, 1 homicide, and 2 undetermined deaths), the *α*-PVP PM blood concentration ranged from <0.02 mg/L to 2 mg/L, but only three cases showed blood concentrations higher than the concentration detectable in our case (0.91 mg/L, 1.3 mg/L, 2.0 mg/L). Only two cases were classified as accidental poisoning due to *α*-PVP alone (2.0 mg/L; 0.04 mg/L). In the latter case, intravenous use of *α*-PVP led to multiple organ damage and eventually death at hospital. At the time of sampling, some time had elapsed from the administration of *α*-PVP to death, which explains the low concentration of the drug. In addition to the cases in which *α*-PVP was the primary intoxicant, 14 cases, excluding the present one, involved *α*-PVP at a fatal multi-drug intoxication level (blood concentrations up to 0.64 mg/L).

The medical literature reports relatively few data on fatal *α*-PVP poisonings. A European Union survey involving eight state members reported 115 deaths between 2012 and 2015 in which *α*-PVP was analytically confirmed in one or more biological samples [25]. In 23 of these cases, *α*-PVP was the cause or a contributing cause of death, and in 5 of these cases, it was the only drug detected. In a sudden death during restraint of an individual with symptoms of excited delirium after self-administration of *α*-PVP, the PM blood concentration was 0.41 mg/L [26], whereas in two other fatal *α*-PVP poisonings, the concentrations were 0.17 mg/L and 0.85 mg/L [27, 28]. A US study has reported three fatal *α*-PVP poisonings with a PM blood concentration of 0.033, 0.054, and > 20 mg/L, the last one occurring in custody after the victim swallowed the drug [29]. As for non-fatal concentrations, in a series of 18 subjects suspected of driving under the influence of drugs, the blood concentration of *α*-PVP ranged from 0.03 to >0.09 mg/L, but only 4 drivers reported side-effects consistent with those of synthetic cathinones [29].

To the best of our knowledge, although amphetamine and other illicit drugs have been sporadically detected in victims of sauna death [30], neither AADs in a sauna nor deaths in a sauna associated with use of *α*-PVP have been reported thus far.

In the present case, the sauna was electrical (as is generally the case in saunas located in residence buildings in Finland), and its heating was interrupted by a timer at the end of the final shift. As Finnish saunas are heated to 60–80 °C or more [31, 32], and the victim’s mother was sauna-bathing until 9:30 p.m. when the victim arrived at the sauna, the victim was likely exposed to significant heat prior his death, although the sauna had significantly cooled by the time the police arrived at the scene.

During a Finnish sauna (temperature 80–100 °C, humidity 10–20%) the skin temperature rises within a few minutes to 40 °C, and the core temperature rises approximately 1 °C in 30 min, while the heart rate increases; heat is lost by increased cutaneous blood flow, vasodilatation, and sweating [32]. In most sauna deaths, a pre-existing cardiac disease or acute alcohol consumption, or both, represent the underlying or contributing cause of death [30, 31]. Exposure to sauna heat may also alter drug pharmacokinetics, especially for trans-dermally administered drugs such as nitroglycerine [32] and fentanyl (unpublished case). Medications and illicit drugs having a thermogenic effect (e.g. amphetamine-like drugs including synthetic cathinones), when associated with exposure to overheated environments and coupled with inadequate liquid intake, can impair body thermoregulatory mechanisms and lead to life-threatening hyperthermia [20, 33, 34]. Body temperatures up to 43.3 °C have been associated with such events as the use of MDMA (“ecstasy”) [33].

In the present case, the thermogenic effects of *α*-PVP and amphetamine, combined with sauna heat, likely caused a marked elevation of the victim’s body temperature. However, no PM diagnosis of hyperthermia was possible. Establishing hyperthermia as the cause of death at autopsy is indeed challenging, because changes are unspecific and occasional (brain and lung edema; cutaneous, pleural, pericardial petechiae), information on ambient temperature and on the victims’ rectal temperature at the scene is hardly available, and retrieval of victims in a sauna is often delayed. Diagnosis of death due to hyperthermia thus remains mostly one of exclusion, based on circumstantial and individual factors and the exclusion, at autopsy, of other causes of death [35–38].

It is likely that the victim described here took *α*-PVP before his sauna shift, while planning to engage in paraphilic activities, possibly foreseeing the drugs effects on heightening his sexual arousal. We are unaware whether this was the first time the victim had consumed drugs and engaged in sexual activities while having a sauna. The sympathomimetic effects of *α*-PVP and amphetamine, testified by the detection of myocardial contraction band necrosis [39] and the additive effects of *α*-PVP and amphetamine with sauna heat on the cardiovascular system and of dehydration, combined with an increasing body core temperature seemingly contributed to the fatal outcome. Moreover, respiratory depression caused by buprenorphine and enhanced by benzodiazepines, as well as the high levels of adrenaline associated with sexual activity, evoked by the sexual props found at the scene, may well have contributed to death. Buprenorphine, although found at a fairly high level, was not considered crucial, since the low buprenorphine to norbuprenorphine ratio did not suggest acute poisoning [40]. It can also be noted, as a matter of general prevention, that timer-regulated locking of a sauna door may delay first-aid intervention for solo sauna bathers who experience any acute incapacitating medical condition, although in our case this was not a relevant issue.

In conclusion, this case report highlights the risk of using allegedly safe designer drugs, for instance *α*-PVP, in a sauna, especially in association with other drugs and while engaging in sexual activity.

## Key points

1. Autoerotic accidental deaths (AAD) result from mechanical asphyxia or can involve other types of sexual self-stimulation.
2. We describe a unique case of AADs during a sauna associated with use of α-PVP, amphetamine and other drugs.
3. The additive cardiovascular effects of α-PVP and amphetamine, sauna heat and dehydration contributed to the fatal outcome.
4. In saunas, using sympathomimetic and other drugs, especially while engaging in sexual activity can be life-threatening.
